# A Man with Severe Right Knee Pain

**DOI:** 10.5811/cpcem.2019.1.41417

**Published:** 2019-02-26

**Authors:** Shinsuke Takeda, Katsuyuki Iwatsuki, Yosuke Takeichi, Tomohiro Kano, Akihiko Tabuchi, Hitoshi Hirata

**Affiliations:** * Anjo Kosei Hospital, Emergency and Critical Care Center, Anjo, Japan; † Anjo Kosei Hospital, Department of Orthopaedic Surgery, Anjo, Japan; ‡ Nagoya University Graduate School of Medicine, Department of Hand Surgery, Nagoya, Japan

## CASE PRESENTATION

**Image 1:**
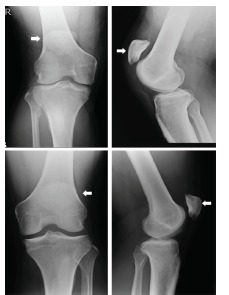
High-riding patella (patella alta) (arrows): (upper) right knee and (lower) left knee. The Insall-Salvati ratio (length from the tibial tubercle to the inferior patellar pole divided by the length of the patella), as seen on the lateral view radiograph, is 1.86 in the right knee and 1.60 in the left knee. (Ratio >1.2 is defined as patella alta.)

**Image 2:**
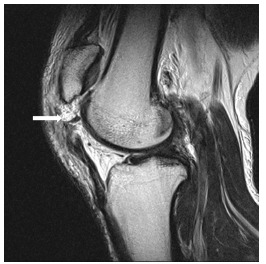
Magnetic resonance sagittal T2-weighted image shows proximal patellar tendon rupture (enthesis of the patella) (arrow).

A 29-year-old man with no significant medical history presented to the emergency department with severe pain, swelling, and inability to move his right knee. He was injured when he extended his right knee to hit a tennis ball after running to the net. On examination, high-riding patellae were found on both the injured and non-injured sides. A lateral view radiograph showed patella alta in both knees (Image 1). Magnetic resonance imaging (MRI) was performed to examine the right knee extensor apparatus (Image 2).

## DISCUSSION

Patellar tendon rupture is an uncommon clinical presentation that generally affects patients younger than 40 years who actively engage in sporting activities.^1^ Patellar tendon rupture from indirect injury in an athlete represents the end stage of jumper’s knee and results from repetitive microtrauma.^2^ It occurs most frequently in patients with predisposing factors such as rheumatoid arthritis, chronic renal failure, systemic lupus erythematosus, hyperparathyroidism, hereditary disorders of the connective tissue (e.g., Ehlers-Danlos syndrome), or long-term medication such as corticosteroids or fluoroquinolones.^1^,^3^,^4^ Because MRI is not always immediately available, the emergency physician should confirm the disrupted extensor mechanism such as loss of active extension of the leg in addition to swelling in the anterior aspect of the knee and hemarthrosis. Patellar tendon rupture occurring in a patient with patella alta is quite rare.^2^ In this case, diagnosis based on radiographs was difficult because the non-injured side also showed patella alta. Patella alta may contribute to or initiate chondromalacia.^2^ After surgery, the patient returned to his baseline level of activity with no complaints.

**CPC-EM Capsule**

What do we already know about this clinical entity?

*Patellar tendon rupture is an uncommon clinical presentation. Patellar tendon rupture occurring in a patient with patella alta is quite rare*.

What is the major impact of the image(s)?

*Diagnosis based on radiographs is difficult because the non-injured side also showed patella alta in this case*.

How might this improve emergency medicine practice?

*Emergency doctors should suspect patella tendon rupture from the clinical findings. Considering our case such as non-injured side also showing patella alta, magnetic resonance imaging is essential for diagnosis*.
